# Airway pressure release ventilation restores hemodynamic stability in patients with cardiogenic shock: initial experience in cardiac intensive care

**DOI:** 10.1186/cc13472

**Published:** 2014-03-17

**Authors:** A Taha, A Shafie, M Mostafa, H Hon, R Marktanner

**Affiliations:** 1 Sheikh Khalifa Medical City, Abu Dhabi United Arab Emirates

## Introduction

Airway pressure release ventilation (APRV) is a pressure- limited, time-cycled mode of mechanical ventilation. It increases the cardiac index, resulting in improved organ perfusion, which is crucial in cardiogenic shock patients preventing organ failure secondary to inadequate perfusion [1]. The purpose of this study was to evaluate the effectiveness of APRV in restoring hemodynamic stability and improving oxygenation in ventilated patients with cardiogenic shock and severe progressive hypoxemia.

## Methods

All cardiac and cardiac surgical patients with cardiogenic shock and ALI/ARDS admitted to our ICU were enrolled between January 2012 and September 2013. Data were collected on admission while the patients were on the conventional mode of ventilation and after 48 hours from switching to APRV. All enrolled patients were hemodynamically monitored with a pulmonary artery catheter and frequent echocardiography assessment. A retrospective analysis of these data was performed.

## Results

Completed datasets were obtained from 29 patients. The cardiac index was increased by 30% (*P *< 0.013), serum lactate decreased by 37% (*P *< 0.001), central venous saturation increased by 42% (*P *< 0.001) and peak airway pressure decreased 19% (*P *< 0.001), with 50% increase of mean airway pressure, hypoxemia improved within the first few hours of alveolar recruitment with PaO_2_/FIO_2 _increased by 23% (*P *< 0.018), and there was less use of vasopressors, sedation and neuromuscular blockage over the course of APRV application.

## Conclusion

In our patient series, APRV significantly improved oxygenation and allowed for spontaneous breathing and a reduction in peak airway pressures. Furthermore, this strategy improved hemodynamics and facilitated weaning from MV. Therefore, our data suggest that this ventilation modality has favorable results and appears to be an effective alternative for lung recruitment in patients with cardiogenic shock and acute lung injury during their course of stay in cardiac ICU [2].
